# Genome wide association mapping and haplotype analysis identify genetic loci and candidate genes associated with yield related traits in rice (*Oryza sativa* L.)

**DOI:** 10.3389/fpls.2026.1909754

**Published:** 2026-09-14

**Authors:** Nansheng Wang, Muhammad Ahmad Hassan, Kang Li, Kunneng Zhou, Quan Gan, Jiafa Xia, Cuixiang Lin, Zefu Li, Fengshun Song, Dahu Ni

**Affiliations:** 1 Rice Research Institute, Anhui Academy of Agricultural Sciences, Hefei, China; 2 College of Resource and Environment, Anhui Agricultural University, Hefei, China; 3 Key Laboratory of Rice Genetics and Breeding in Anhui Province, Hefei, China

**Keywords:** candidate genes, Genome Wide Association Studies (GWAS), grain yield, haplotype analysis, quantitative trait loci (QTLs), rice, yield related traits

## Abstract

Rice (*Oryza sativa* L.) is one of the most important staple crops worldwide, and improving grain yield remains a major objective of rice breeding programs. Yield is a complex quantitative trait influenced by multiple agronomic characteristics, including grain yield per plant (GY), seed setting rate (SSR), plant height (PH), and thousand grain weight (TGW). In this study, a natural population comprising 265 rice accessions was evaluated during two consecutive growing seasons (2022-2023) at one field location, and genome-wide association studies (GWAS) conducted using 4454137 high-quality single nucleotide polymorphisms (SNPs). Best linear unbiased prediction (BLUP) values estimated across the two year-environments were used for association analysis using a mixed linear model (MLM). A total of 102 significant SNPs were identified and consolidated into 20 quantitative trait loci (QTLs), including five associated with GY, four with SSR, ten with PH, and one with TGW. Among them, several loci were co-localized with previously reported genes associated with yield related traits, thereby supporting the reliability of the GWAS results. Candidate gene identification and haplotype analysis further revealed four prioritized genes: *LOC_Os09g28230 (GID1L2)* for GY, *LOC_Os05g09500* for SSR, *LOC_Os03g06930* for PH, and *LOC_Os02g09170* for TGW. Significant phenotypic differences among haplotypes supported the potential involvement of these genes in regulating the natural trait variation. These findings provide insights into the genetic basis of rice yield related traits and offer genetic resources for future validation and breeding. However, because the present study included only two year-environments at one field location, additional multi-environment validation is required before the broader environmental reproducibility or breeding adaptation.

## Introduction

Food security is fundamental to human survival, economic development, and social stability (Hickey et al., 2019). Rice (*Oryza sativa* L.) serves as the staple food for more than half of the global population and contributes more than 20% of the daily caloric intake of nearly 4 billion people worldwide (Hassan et al., 2023; Mohidem et al., 2022). As the global population is projected to approach 10 billion by the middle of this century (Lam, 2025), food demand is expected to increase substantially (Hickey et al., 2019). To meet this challenge, global food production must increase by 50% compared with current levels. However, rapid urbanization, shrinking arable land resources, and increasingly frequent climate-related stresses, including drought and high temperature events, pose significant threats to agricultural productivity (Chen et al., 2022). Therefore, enhancing rice yield within existing cultivation areas has become a critical objective for ensuring future food security.

Grain number per panicle (GNP), number of effective panicles per plant and thousand-grain weight (TGW) are major determinants of rice yield, and each of these components is influenced by multiple underlying agronomic traits (Li et al., 2021; Hu et al., 2025; Zhang et al., 2022). For example, the number of filled grains per panicle depends on both the total number of spikelets per panicle and the seed setting rate (SSR). SSR, defined as the proportion of the number of filled grains relative to the total number of spikelets, is an important determinant of the rice productivity (Li et al., 2021). As one of the most environmentally sensitive yield related traits, SSR is strongly affected by the environmental factors, particularly high-temperature and drought stress (Hu et al., 2023). In addition, SSR depends on the multiple reproduction developmental processes, including pistil and stamen development, fertilization, and grain formation (Yin et al., 2022; Hong et al., 2022 ;Wang et al., 2023; Song et al., 2018). Another development and pollen fertility are critical determinants of the SSR. For example, mitochondrial ribosomal protein gene *OsMRPL15* regulates pollen fertility by influencing starch biosynthesis in pollen grains, thereby affecting SSR (Xie et al., 2023). Anther dehiscence directly influences pollen release and fertilization efficiency. The kinesin-like protein PSS1 participates in the regulation of anther dehiscence and contributes to successful fertilization in rice (Zhou et al., 2010), whereas *SEC3A* regulates anther dehiscence through the Auxin signaling pathway and influences the SSR (Tu et al., 2024). Furthermore, successful pollen germination and pollen tube elongation are essential for fertilization, *OsCNGC13* promotes pollen tube growth within stylar tissues and thereby contributes to the regulation of SSR (Xu et al., 2017). Although numerous genes associated with SSR have been identified, the genetic basis underlying natural variation in this trait remains incompletely understood.

The TGW and grain yield per plant (GY) are important indicators of rice productivity and are primarily influenced by the grain size and grain-filling efficiency (Zhang et al., 2023). To date, numerous quantitative trait loci (QTLs) associated with grain weight have been identified, although only a limited number have been functionally characterized and cloned (Wang et al., 2024). Among these QTLs, *GS2*, which encodes the transcription factor *OsGRF4*, positively regulates grain size and grain weight, and favorable alleles significantly increase grain yield per plant (Chen et al., 2016; Duan et al., 2015; Hu et al., 2015; Li et al., 2016). *TGW6*, located on chromosome 6, encodes an IAA-glucose hydrolase, and introgression of its favorable allele from Kasalath into Nipponbare increases GY by up to 15% (Ishimaru et al., 2013). Another major grain weight QTL, *GW2*, encodes a RING-type protein with E3 ubiquitin ligase involved in grain development, and loss of function mutations in this QTL significantly increases grain width, grain weight, and GY (Song et al., 2007; Yan et al., 2011; Huang et al., 2022). In addition, *miR5504* regulates grain width and grain thickness through modulation of its target gene *LOC_Os08g16914*, thereby influencing grain weight and GY (Wang et al., 2024). The large-grain gene BG1 promotes grain development by modulating the auxin transport (Liu et al., 2015), whereas *GSN1* (*OsMKP1*) regulates grain size through the *OsMKKK10-OsMKK4-OsMAPK6* signaling cascade that controls glume cell proliferation (Guo et al., 2018). Collectively, these studies demonstrate that TGW is regulated by diverse biological pathways, including hormone signaling transcriptional regulation, ubiquitin-mediated protein degradation, and developmental signaling networks. Therefore, many loci controlling grain weight and yield remain undiscovered, highlighting the importance of further genetic dissection using diverse rice germplasm resources (Zhang et al., 2023).

Plant height (PH) is a key agronomic trait in rice that influences the plant architecture, lodging resistance, biomass accumulation, and grain yield. Appropriate plant height contributes to the improved canopy structure and light interception efficiency, thereby supporting biomass production. However, excessive PH increases susceptibility to lodging, which can reduce grain yield and production stability. Therefore, identifying favorable alleles that optimize PH and plant architecture remains an important objective in rice breeding. To date, scientists have identified and characterized more than 70 genes associated with PH regulation and dwarfism have been identified and functionally characterized in rice. Among these genes, *SBI* encodes a gibberellin 2-oxidase (GA2-oxidase) involved in gibberellin (GA) metabolism and plays an important role in regulating internode elongation (Li et al., 2019). *OsBRI1*, which encodes a brassinosteroid (BR) receptor kinase, is a key component of BR signaling, and its mutant *d61* exhibits dwarfism and erect leaves (Gao et al., 2014). The *D53* gene encodes a class I ClpATPase-like protein, involved in strigolactone (SL) signaling, and dominant mutation in this gene, resulted in a dwarf and high tillering phenotype that is intensive to SL (Tal et al., 2022). The “Green Revolution” gene *sd-1*, which encodes GA 20-oxidase, is one of the most influential genes in rice breeding history. Mutations in *sd-1* reduce GA biosynthesis, resulting in a semi-dwarf plant with enhanced lodging resistance and improved harvest index, thereby contributing substantially to global increases in rice productivity during the Green Revolution (Monna et al., 2001). In brief, these studies demonstrate the PH is regulated by multiple genetic pathways, including GA, BR, and SL signaling (Tal et al., 2022). Therefore, substantial natural variation controlling PH remains to be explored in diverse rice germplasm resources.

Although numerous QTLs and genes associated with GY, SSR, PH, and TGW have been identified, the genetic architecture underlying these complex traits remain incompletely understood because of their polygenic inheritance and strong environmental influences. Despite substantial progress in rice functional genomics, many favorable alleles contributing to the natural variations in yield related traits remain undiscovered, particularly in diverse germplasm resources (Chen et al., 2022). The integration of high density GWAS with haplotype analysis provides an effective approach for identifying candidate genes and elite alleles that can be utilized in molecular breeding programs (Yano et al., 2016; Huang and Han, 2014). To further dissect the genetic basis of yield related traits, a diverse natural population comprising 265 rice accessions provided by the Rice Research Institute of the Anhui Academy of Agricultural Sciences was investigated. Using 4454137 high-quality single nucleotide polymorphisms (SNPs) and phenotypic data collected over two consecutive years, GWAS was performed to identify loci associated with GY, SSR, PH, and TGW. Furthermore, candidate gene identification and haplotype analysis were conducted to prioritize promising genes underlying trait variation and favorable alleles for future marker-assisted selection (MAS) and molecular breeding. In this study, materials provided by the Rice Research Institute of the Anhui Academy of Agricultural Sciences were used as research subjects. By combining high-throughput sequencing, we performed repeated multi-year QTL mapping for traits affecting single plant yield, seed setting rate, plant height, and thousand-grain weight in the population. Additionally, we explored and functionally analyzed important candidate genes, aiming to provide a theoretical foundation for breeding high-yielding and superior rice varieties. The findings of this study provide valuable insights into the genetic mechanisms controlling rice yield related traits and offer useful genetic resources for the development of high yielding rice cultivars.

## 2 Materials and methods

### 2.1 Plant material and growth conditions

A natural population consisting of 265 rice accessions (**Supplementary Table 1**), collected, maintained and provided by the Rice Research Institute of Anhui Academy of Agricultural Sciences, was used in this experiment. Field experiments were conducted during 2022 and 2023 growing seasons at the Rice Research Base of the Rice Research Institute, Anhui Academy of Agricultural Sciences, located in Xingfu Village, Shucheng County, Lu’an City, Anhui Province. Rice seedlings at 25 to 30 days after sowing (DAS) were transplanted into the field. Manual transplantation was performed using a single seedling per hill with a row spacing of 20 cm, and a plant spacing of 25 cm. The field experiment was planted in a randomized complete block design (RCBD) with three field replicates. In each replication, each rice accession was planted in five rows with ten plants per row. Standard agronomic management practices for rice cultivation were applied throughout the growing season. All accessions were grown under the same field conditions to minimize the environmental variation.

### 2.2 Phenotypic evaluation and statistical analysis

The PH was measured at the maturity stage when approximately 60% of the panicles reached yellow ripening. The PH was determined manually from the soil surface at the base of the plant to the tip of the tallest panicle. To minimize the border effects, plants located in the outer rows were excluded from the phenotypic evaluation. Within each replication, five representative plants with uniform growth were randomly selected from the central rows of each accession, and the mean value was used for subsequent statistical analysis.

Following PH assessment, three representative plants from each accession within each replication were selected for yield-related trait evaluation. Mature grains were harvested, mixed, and dried before further measurements. Due to the variations in growth periods of rice accessions, they were harvested at different times. The GY was determined by weighing the harvested grains from each selected plant after drying. For TGW determination, 200 filled grains were randomly selected and weighed in triplicate and then averaged for TGW of each rice accession. The SSR was calculated as the ratio of filled grains to the total number of spikelets and expressed as percentage. Trait evaluation was conducted according to the standard evaluation system for rice (SES) developed by international rice research institute (International Rice Research Institute, 2013).

Microsoft Excel and R studio were used for data processing and statistical analysis. Descriptive statistics, including mean, standard deviation (SD), minimum, maximum, coefficient of variation (CV), and broad sense heritability (H_B_^2^) (Nyquist and Baker, 1991), were calculated for each trait. Correlation analysis among traits were performed using two-year phenotypic data (Benesty et al., 2009). Frequency distribution histograms were generated using Origin 2020 software.

Broad-sense heritability (H_B_^2^) was estimated using variance components obtained from the mixed-model analysis of the phenotypic data collected across the two growing seasons. The H_B_^2^ was calculated as follows:


$${\text{H}}_{\text{B}}^{2}=\frac{Vg}{Vg+\frac{V\epsilon }{l}}$$

where, *Vg* represents the genetic variance, *Vϵ* represents the residual variance, and *l* represents the number of environments.

Broad-sense rather than narrow-sense heritability was estimated because the objective of this study was to evaluate the overall genetic contribution to phenotypic variation of diverse rice accessions across the two year-environments, whereas estimation of narrow-sense heritability requires portioning additive genetic variance, which was beyond the scope of the present study.

Phenotypic data obtained from three replicates in each environment were averaged for descriptive statistical analysis. To account for environmental variation between the two growing seasons and improve estimation of genetic effect variation, BLUP values were subsequently calculated across the two year-environments and used for the genome wide association study (GWAS).

### 2.3 Genome-wide association analysis and candidate gene identification

Phenotypic data collected across 2022 and 2023 growing seasons were used to estimate BLUP values using Ime4 package in R (Bates et al., 2014; Piepho et al., 2008). The BLUP values and SNP genotype data obtained from the rice population were subsequently subjected to GWAS using a mixed linear model (MLM) in TASSEL software (Bradbury et al., 2007; Yu et al., 2006; Kazemzadeh et al., 2025). The genotype dataset consisted of 4454137 high-quality SNPs retrieved from the publicly available whole-genome resequencing dataset of the 265 rice accessions used in this study. The resequencing data have been deposited in the NCBI BioProject database under accession number PRJNA1141731 (Li et al., 2024). Detailed procedures for the whole genome resequencing, sequence alignment, SNP calling, quality control filtering, genotype imputation, and population genetic analysis have been described in our research group’s previous study (Li et al., 2024). The MLM accounts for both population structure and kinship relationships, thereby reducing the occurrence of false-positive associations. The proportion of phenotypic variance explained (PVE) for each significant QTL was obtained from the MLM implemented in TASSEL and presented in **Table 1**. The effective number of independent tests was estimated using the genetic type 1 error calculator (GEC) method (Li et al., 2012). Because highly stringent genome wide correction may increase the risk of false negatives in complex trait analysis, a practical genome-wide significant threshold was determined based on the effective number of independent tests (Me) estimated by GEC following the method of Li et al. (2012). Accordingly, significance threshold was calculated as 0.05/Me resulting in a genome wide significance threshold of *p* < 6.74 × 10^-6^. SNPs with *p* values below this threshold were considered significantly associated with the investigated traits.

**Table 1:** Significant QTLs associated with yield related traits identified by GWAS in 265 rice accessions.

Trait	QTLs	Chr	Lead SNP position (bp)	Physical interval (bp)	*p*-value	PVE (%)	Previously reported genes/QTLs
GY	*qGY1.1*	1	29585993	2758993-3158993	3.77E-07	11.66	*LOGL1* (Sandhu et al., 2024)
	*qGY7.1*	7	9212253	9012253-9412253	5.73E-07	11.74	*Ghd7*; *E1*; *Hd4* (Xue et al., 2008)
							*NAL8* (Chen et al., 2019)
	*qGY9.1*	9	3687556	3487556-3887556	6.53E-07	11.2	*OsbHLH120*; *MOG1* (Han et al., 2024)
	*qGY9.2*	9	17301720	17101720-17501720	6.37E-07	11.57	
	*qGY11.1*	11	5115640	4915640-5315640	3.64E-08	13.63	
SSR	*qSSR5.1*	5	5449803	5249803-5649803	6.26E-08	13.4	
	*qSSR6.1*	6	8451483	8251483-8651483	2.29E-09	16.59	
	*qSSR7.1*	7	3185614	2985614-3385614	3.35E-07	11.99	
	*qSSR8.1*	8	5432293	5232293-5632293	2.38E-07	12.26	
PH	*qPH1.1*	1	5174943	4974943-5374943	4.35E-07	12.35	*DPF*; *OsbHLH025* (Yamamura et al., 2015)
							*D2*; *CYP90D2*; *smg11* (Dong et al., 2016)
							*OsRDR3* (Jha et al., 2021)
	*qPH1.2*	1	25853585	25653-585-26053585	2.80E-07	12.14	*SLRL1* (Itoh et al., 2005)
	*qPH1.3*	1	39129706	38939706-39329706	4.86E-07	11.67	*Os1BGlu4*; *Os1βGlu4* (Xu et al., 2021)
	*qPH3.1*	3	3399654	3199654-3599654	1.76E-07	12.53	
	*qPH3.2*	3	13361063	13161063-13561063	2.77E-07	12.43	*OsCHR732*; *OsINO80* (Li et al., 2018)
	*qPH4.1*	4	482111	682111-282111	2.27E-07	13.51	*OsARG* (Ma et al., 2012)
	*qPH6.1*	6	24726936	24526936-24926936	2.97E-07	12.17	
	*qPH9.1*	9	11658219	11458219-11858219	3.10E-08	14.27	*OsCBM1* (Jing et al., 2021)
	*qPH9.2*	9	18057440	17857440-18257440	2.72E-08	14.14	*OsISA3* (Wang et al., 2023)
							*Oshox4* (Dai et al., 2008)
							*OsBIM1*; *OsbHLH032* (Tian et al., 2021
	*qPH11.1*	11	13254845	13054845-13454845	2.99E-07	12.08	
TGW	*qTGW2.1*	2	4737804	4537804-4937804	2.29E-07	12.72	*gh2*; *OsCAD2* (Wang et al., 2017)

Candidate QTL intervals were defined as the ± 200 kb around each lead SNP based on the linkage disequilibrium decay distance of the study population. The chromosomal distribution of all identified QTLs and their co-localized previously reported genes/QTLs is shown in **Supplementary Figure 1**.

Within each significant genomic region, the SNP with the lowest *p* value was designated as the lead SNP representing the QTL. Candidate regions were defined as 200 kb upstream and downstream of each lead SNP based on the linkage disequilibrium (LD) decay distance; previous analysis of the same germplasm population showed that LD decays over approximately 100-300 kb (Li et al., 2025); therefore a ± 200 kb interval was adopted for candidate gene identification, consistent with the observed LD decay and previous rice GWAS studies (Xiao et al., 2019; Huang et al., 2010). Genes located within the candidate intervals were retrieved from the “Rice Genome Annotation Project Database (http://rice.plantbiology.msu.edu)”, and comprehensively catalogued. Candidate gene prioritization was performed using multiple complementary criteria, including functional annotation relevant to the investigated trait, presence of nonsynonymous variants in coding regions, available expression or functional evidence from public rice genomic resources and published studies, and consistency of haplotype-phenotype associations across the two growing seasons. Genes that did not meet sufficient evidence for prioritization were retained in the comprehensive annotation dataset (**Supplementary Table 4**) but were not subjected to detailed haplotype analysis.

SNPs located within candidate genes and their promoter regions were extracted from the genome wide SNP data set for haplotype analysis. Functional annotation of SNPs was performed using SnpEff based on Nipponbare reference genome (http://rice.plantbiology.msu.edu/cgi-bin/gbrowse/rice/) (Cingolani et al., 2012), particularly focused on non-synonymous SNPs with potential functional effects on protein coding sequences. Heterozygous and missing alleles were excluded from the subsequent analysis, and haplotypes represented by fewer than 10 accessions were removed to ensure statistical reliability. Phenotypic differences among haplotypes were evaluated using analysis of variance (ANOVA) followed by Tukey’s multiple comparison test (Tukey, 1949).

To further support candidate gene prioritization, publicly available transcriptome datasets were used to evaluate the expression profiles of the selected candidate genes. Expression information was obtained from the Rice Expression Database (RED), and tissues and developmental stages associated with yield-related traits were considered. The expression profiles of the 4 prioritized candidate genes were summarized in **Supplementary Table 5**.

## 3 Result

### 3.1 Phenotypic variation and correlation analysis of yield-related traits

Grain yield is a major target trait in rice breeding, and its formation is influenced by multiple agronomic components such as GY, SSR, PH and TGW. Characterizing phenotypic variation and trait correlation is essential for understanding their genetic basis. Phenotypic evaluation of 265 rice accessions across two consecutive years revealed substantial variations among all investigated traits (**Table 2**). The mean GY values were 20.01 ± 7.5 g in 2022, and 26.68 ± 6.86 g in 2023, with ranges of 2.65-39.93 g and 6.20-45.67 g, respectively. Significant differences in trait distributions were observed between years, indicating a strong environmental influence on GY performance. The mean SSR increased from 63.68 ± 16.46% in 2022 to 85.01 ± 8.56% in 2023, demonstrating considerable year-to-year variation. In contrast, PH and TGW showed relatively similar distributions across years. The frequency distribution of PH and TGW were concentrated within the ranges of 80-100 cm and 21-24 g, respectively, and both traits exhibited CV below 20% (**Table 2**; **Figure 1**). The H_B_^2^ estimates ranged from 52.40% to 94.72%, indicating moderate to high genetic control of the traits investigated. Overall, the observed phenotypic variation supports the suitability of this population for genetic analysis and association mapping.

**Table 2:** Descriptive statistics of yield-related traits in 265 rice accessions.

Trait	Year	Mean ± SD	Range	CV (%)	H_B_^2^ (%)
GY	2022	20.01 ± 7.5	2.65-39.93	37.49	73.09
	2023	26.68 ± 6.86	6.2-45.67	25.7	
SSR	2022	63.68 ± 16.46	9.74-97.7	25.84	52.4
	2023	85.01 ± 8.56	28.07-96.46	10.06	
PH	2022	105.53 ± 20.01	59.1-191.5	18.96	94.72
	2023	113.32 ± 19.01	73.3-190.2	16.78	
TGW	2022	23.78 ± 3.41	12.4-32.4	14.36	94.5
	2023	24.81 ± 3.45	14-35.6	13.92	

**Figure 1:**
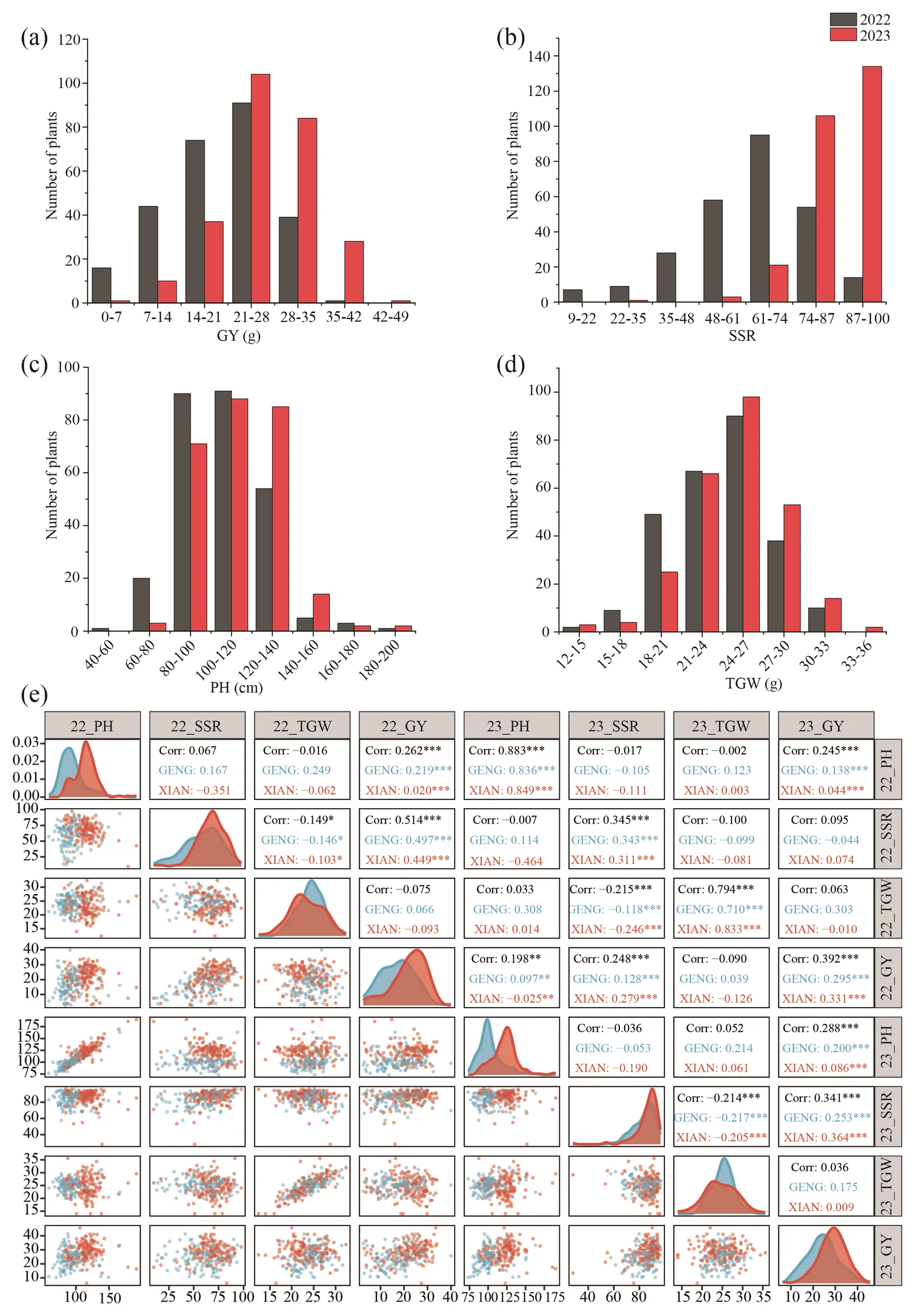
Phenotypic distributions and pairwise correlations of the four agronomic traits evaluated in the 265 rice accessions during 2022 and 2023 growing seasons. **(a–d)** Frequency distribution of grain yield per plant (GY), seed setting rate (SSR), plant height (PH), and thousand grain weight (TGW), respectively, in 2022 and 2023. The x-axis indicates the trait intervals and y-axis represents the number of accessions within each interval. **(e)** Pairwise relationship among the four traits in the two growing seasons. The diagonal panels show the phenotypic distribution (density curves of each trait), the lower triangular panels present scatter plot illustrating pairwise relationship among traits, and the upper triangular panels display Pearson’s correlation coefficients. Correlation coefficients for the entire population (Corr), are shown in black, whereas those for *japonica* (*Geng*) and *indica* (*Xian*) subpopulations are shown in blue and red, respectively. Here, * indicates statistical significant correlations (*p < 0.05*, *p < 0.01*, *p < 0.001*); GY (g plant^-1^), SSR (%), PH (cm), and TGW (g).

Correlation analysis showed that PH exhibited the strongest inter-year correlation (r = 0.883, *p <* 0.001), indicating high consistency of PH measurements between the two evaluated years (**Figure 1**). In contrast, GY showed a relatively weak inter-year correlation (r = 0.245, *p* < 0.001), whereas SSR exhibited little consistency between years (r = − 0. 017), suggesting substantial environmental effects on these traits. Similar correlation patterns were observed in both *XIAN* (*indica*) and *GENG* (*japonica*) subpopulations. GY was positively correlated with SSR (r = 0.514, *p* < 0.001), and PH (r = 0.262, *p* < 0.001), although the relationship between GY and PH was more evident in the GENG subpopulation. Only a weak correlation was detected between GY and TGW. In addition, SSR and PH showed a negative correlation in both subpopulations. Collectively these findings demonstrate substantial phenotypic diversity and complex relationships among yield related traits, providing a solid foundation for subsequent GWAS analysis. Significant differences in the trait distributions between the two growing seasons indicate that year-to-year environmental variation influenced trait performance, particularly for complex traits such as GY and SSR.

### 3.2 Genome-wide association analysis of yield-related traits

In this study, a total of 4,454,137 high-quality SNPs were obtained from the genomic data of 265 rice accessions and used for GWAS of four yield related traits GY, SSR, PH, and TGW. GWAS was performed using MLM that integrated SNP genotypes and phenotypic data. In total, 102 significant SNPs were detected (**Supplementary Table 2**). These SNPs were distributed across 10 of the 12 chromosomes, with no significant association detected on chromosomes 10 and 12. Based on the estimated LD decay distance, adjacent significant SNPs located within 200 kb of each other were grouped into a single QTL. Consequently, 20 QTLs associated with investigated traits were defined (**Table 1**; **Figure 2**).

**Figure 2:**
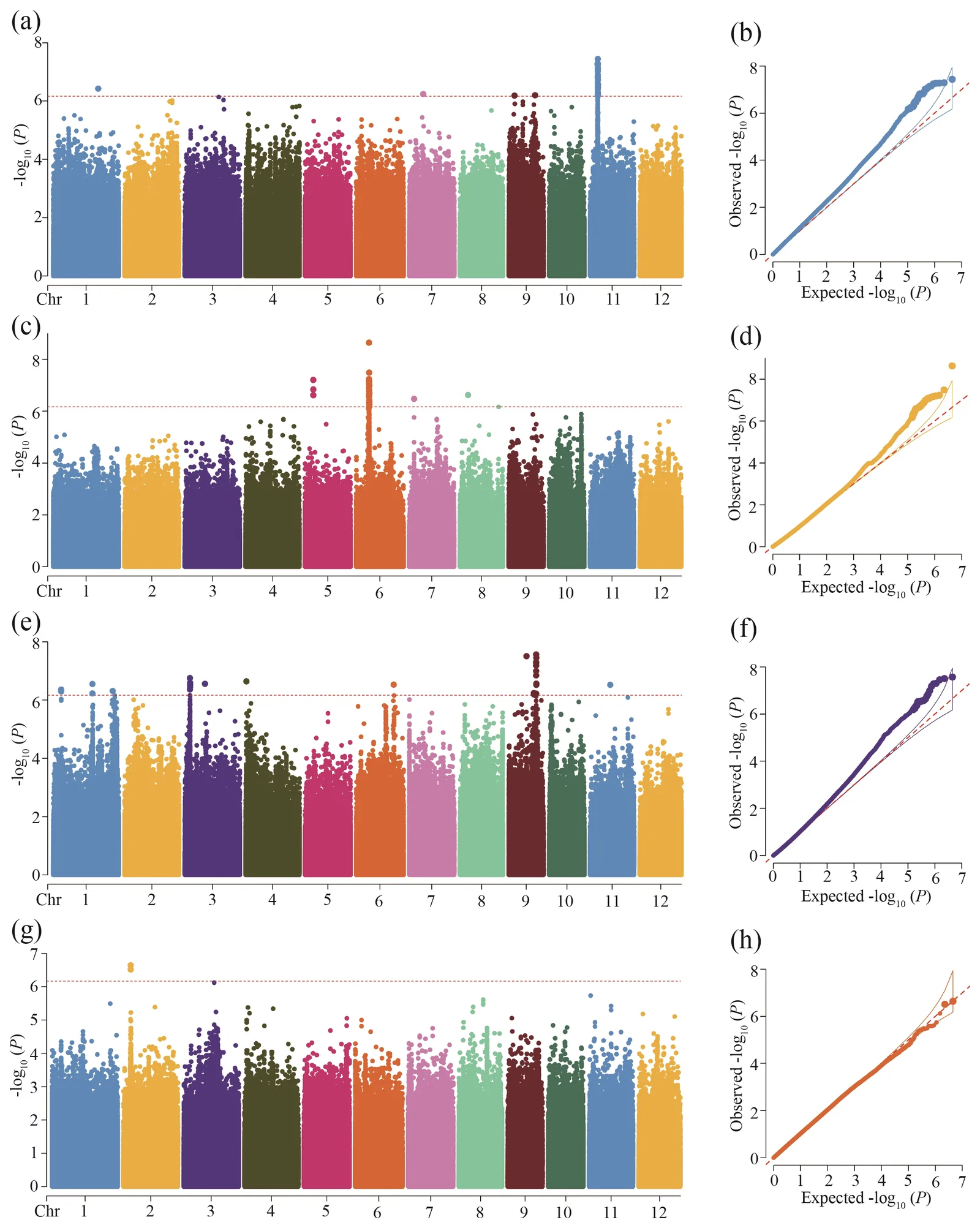
Genome wide association analysis of four yield related traits in 265 rice accessions. **(a, b)** Manhattan and quantile-quantile (Q-Q) plot for grain yield per plant; **(c, d)** seed setting rate; **(e, f)** plant height; **(g, h)** thousand grain weight. The horizontal dashed line indicated the significance threshold (*p* < 6.74 × 10^-6^).

Five QTLs associated with GY were identified on chromosomes 1, 7, 9, and 11. Among them, qGY*11.1* on chromosome 11 exhibited the largest phenotypic effect, explaining 13.63% of the phenotypic variance (PVE), whereas *qGY9.1* on chromosome 9 showed the lowest PVE (11.2%). Several previously characterized yield-related genes, including *LOGL1*, *Ghd7*, *NAL8*, and *MOG1* were located within or near these QTL intervals (*qGY 1.1*, *qGY 7.1*, *qGY 9.1*, *qGY 9.2*, *qGY 11.1*).

Four QTLs associated with SSR were detected on chromosomes 5, 6, 7, and 8. Among these loci, *qSSR6.1* on chromosome 6 exhibited the strongest association signal, explaining the 16.59% of the PVE and displaying the lowest *p*-value of (2.29 × 10^-9^), whereas, *qSSR7.1* on chromosome 7 had the lowest PVE (11.99%) and the highest *p*-value (3.35 × 10^-7^).

PH exhibited the most complex architecture, with ten significant QTLs distributed across chromosomes 1, 3, 4, 6, 9, and 11. Among these loci, *qPH9.1* on chromosome 9 had the highest proportion of PVE (14.27%), whereas, *qPH9.2* exhibited the most significant association signals and showed the lowest *p*-value (2.72 × 10^-8^). *qPH1.2* on chromosome 1 showed the lowest PVE (11.67%) and the highest *p*-value. Multiple previously reported genes, including *DPF*, *D2*, *SLR1*, *OsINO80*, *OsARG*, *OsCBM1*, *OsISA3*, *Oshox4* and *OsBIM1* were located within the identified QTL intervals, near *qPH1.1*, *qPH1.2*, *qPH3.2*, *qPH4.1*, *qPH9.1* and *qPH9.2*.

In comparison to other traits, only one QTL associated with TGW was detected on chromosome 2. The QTL *qTGW2.1* had explained the 12.72% of PVE and a *p*-value of 2.29 × 1^-7^. It contained a previously reported grain-weight related gene *OsCAD2*, providing the additional support for reliability of the association signal.

It is noted that 11 of the 20 identified QTLs were co-localized with previously reported genes or QTLs associated with yield-related traits, whereas the remaining loci may represent novel genomic regions controlling rice yield components. The chromosomal distribution of the of all identified QTLs and their relationship with previously reported genes/QTLs are illustrated in the **Supplementary Figure 1**. These findings demonstrate the effectiveness of the GWAS approach and provide a foundation for subsequent candidate genes identification and haplotype analysis.

### 3.3 Candidate gene identification and haplotype analysis for grain yield per plant

Five significant QTLs associated with GY were identified through GWAS. Candidate genes within all five QTL intervals were annotated and are provided in **Supplementary Table 4**. After excluding genes annotated as hypothetical proteins, retrotransposons, and transposon-related proteins, candidate genes were prioritized according to the functional annotation and sequence variation. Candidate genes containing non-synonymous SNPs were further evaluated through haplotype analysis. Among the 5 GY-associated QTLs, *qGY9.2* (17.1-17.5 Mb) was selected for detailed candidate gene analysis because it contained multiple annotated genes with functional relevance to plant growth and grain production. The other GY-associated QTLs were retained as significant loci and their genes are systematically annotated (**Supplementary Table 4**) but they were not subjected to the same level of candidate gene and haplotype characterization in this study.

Within *qGY9.2* (17.1-17.5 Mb) region, a total of 34 annotated genes were identified, and their gene IDs and functional annotation are provided in **Supplementary Table 4**. Among these genes, *LOC_Os09g28230*, *LOC_Os09g28300*, and *LOC_Os09g28460* contained non-synonymous variants. Candidate prioritization was not based solely on non-synonymous variation; functional annotation, coding variation, haplotype-phenotype associations and publicly available expression information were considered together to identify genes with convergent evidence supporting their potential involvement in GY. *LOC_Os09g28230*, annotated as the gibberellin receptor (*GID1L2*), was therefore prioritized for further analysis.

Haplotype analysis based on the polymorphisms within *LOC_Os09g28230* classified the accessions into three major haplotypes (**Figure 3**). In 2022, the mean GY values of HapA, HapB, and HapC were 22.80 g, 17.70 g and 15.08 g, respectively. In 2023, the corresponding mean values were 28.96 g, 26.06 g, and 20.13 g, respectively. The three haplotypes (HapA, HapB, HapC) were represented by 106, 75, and 11 accessions, respectively. Across the two year-environments, HapA exhibited significantly higher GY values than HapB and HapC. The similar direction of haplotype effect in the two evaluated years supports further evaluation of HapA as a potentially favorable haplotype combination; however additional environments are required to assess its broader stability. Public expression data (obtained from Rice Expression Database) further showed that *LOC_Os09g28230* was expressed in rice panicles, with an expression value of 2.96781 at 7 days after flowering providing additional evidence that the gene is transcriptionally active during reproductive development (**Supplementary Table 5**). In brief, functional annotation, sequence variation, haplotype-phenotype association, and public expression evidence support the prioritization of the *LOC_Os09g28230* as a candidate gene associated with natural variation in GY.

**Figure 3:**
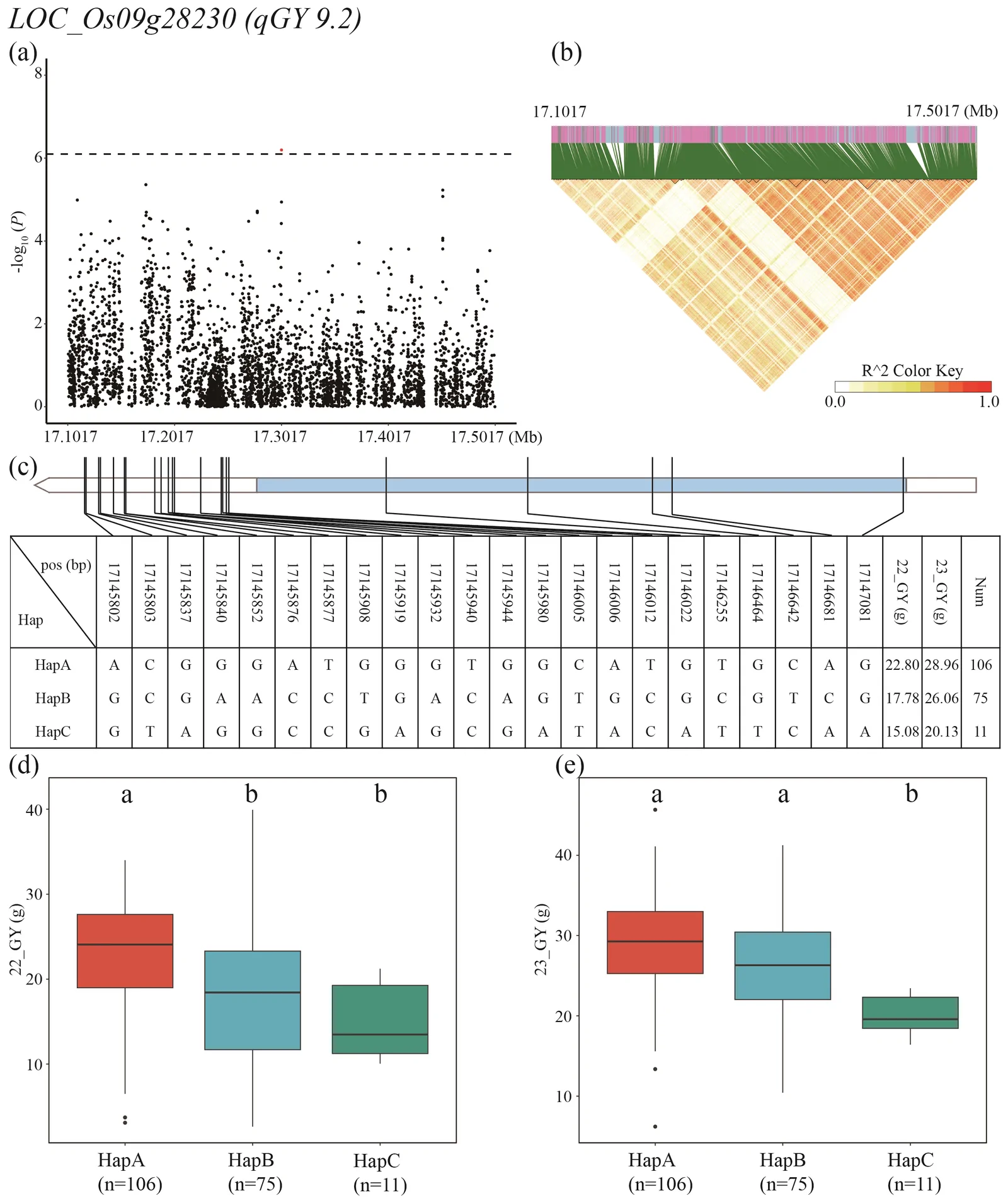
Haplotype analysis of *LOC_Os09g28230* associated with grain yield per plant. **(a)** Regional Manhattan plot of *qGY9.2*; **(b)** linkage disequilibrium heatmap of the candidate region; **(c)** gene structure and haplotype composition of *LOC_Os09g28230*; **(d–e)** comparison of GY among haplotypes in 2022 and 2023. Different letters indicate significant differences among haplotype analysis according to Tukey’s multiple comparison test (*p* < 0.05).

Expression analysis based on public RNA-seq datasets showed that *LOC_Os09g28230* was expressed in reproductive tissues, including panicle and seed tissues (**Supplementary Table 5**). The expression pattern of this gene during reproductive development provides additional evidence supporting its potential involvement in grain yield regulation.

### 3.4 Candidate gene identification and haplotype analysis for seed setting rate

Candidate genes associated with SSR were further investigated within the identified QTL intervals. Candidate genes with all SSR-associated and corresponding gene information is provided in **Supplementary Table 4**. Within the *qSSR5.1* region (5.2–5.6 Mb), a total of 25 annotated genes were identified, and their gene IDs and functional annotation are provided in **Supplementary Table 4**. Among these genes, *LOC_Os05g09380*, *LOC_Os05g09400*, *LOC_Os05g09500*, and *LOC_Os05g09740* contained non-synonymous variants. Candidate prioritization was not based solely on non-synonymous variation; functional annotation, coding variation, haplotype-phenotype association, and publicly available expression information were considered together. Among the genes identified within *qSSR5.1*, *LOC_Os05g09500*, annotated as a cytosolic hexokinase encoding, was prioritized for further analysis because of its functional relevance to sugar metabolism and reproductive development and presence of the informative sequence variation.

Haplotype analysis based on the polymorphisms within *LOC_Os05g09500* classified the accessions into three major haplotypes (**Figure 4**). In 2022, the mean SSR values of HapA, HapB, and HapC were 67.69%, 57.87%, and 57.06%, respectively. In 2023, the corresponding mean values were 85.88%, 84.08%, and 81.55%, respectively. The three haplotypes comprised 126, 54, and 15 accessions, respectively. Across the two year-environments, HapA consistently exhibited significantly higher SSR values than HapB, and HapC, whereas no significant differences were observed between HapB and HapC. The similar direction of haplotype effect in the two evaluated years supports further evaluation of HapA as a potentially favorable haplotype combination; however additional environments are required to assess its broader stability. In addition, public expression data showed that *LOC_Os05g09500* was expressed in rice panicles, with an expression value of 4.88352 at 7 days after flowering (**Supplementary Table 5**), and was also expressed in developing seeds, supporting its transcriptional activity during reproductive and seed development stages. In brief, functional annotation, sequence variation, haplotype-phenotype associations and public expression evidence support the prioritization of the *LOC_Os05g09500* as a candidate gene associated with natural variation in SSR.

**Figure 4:**
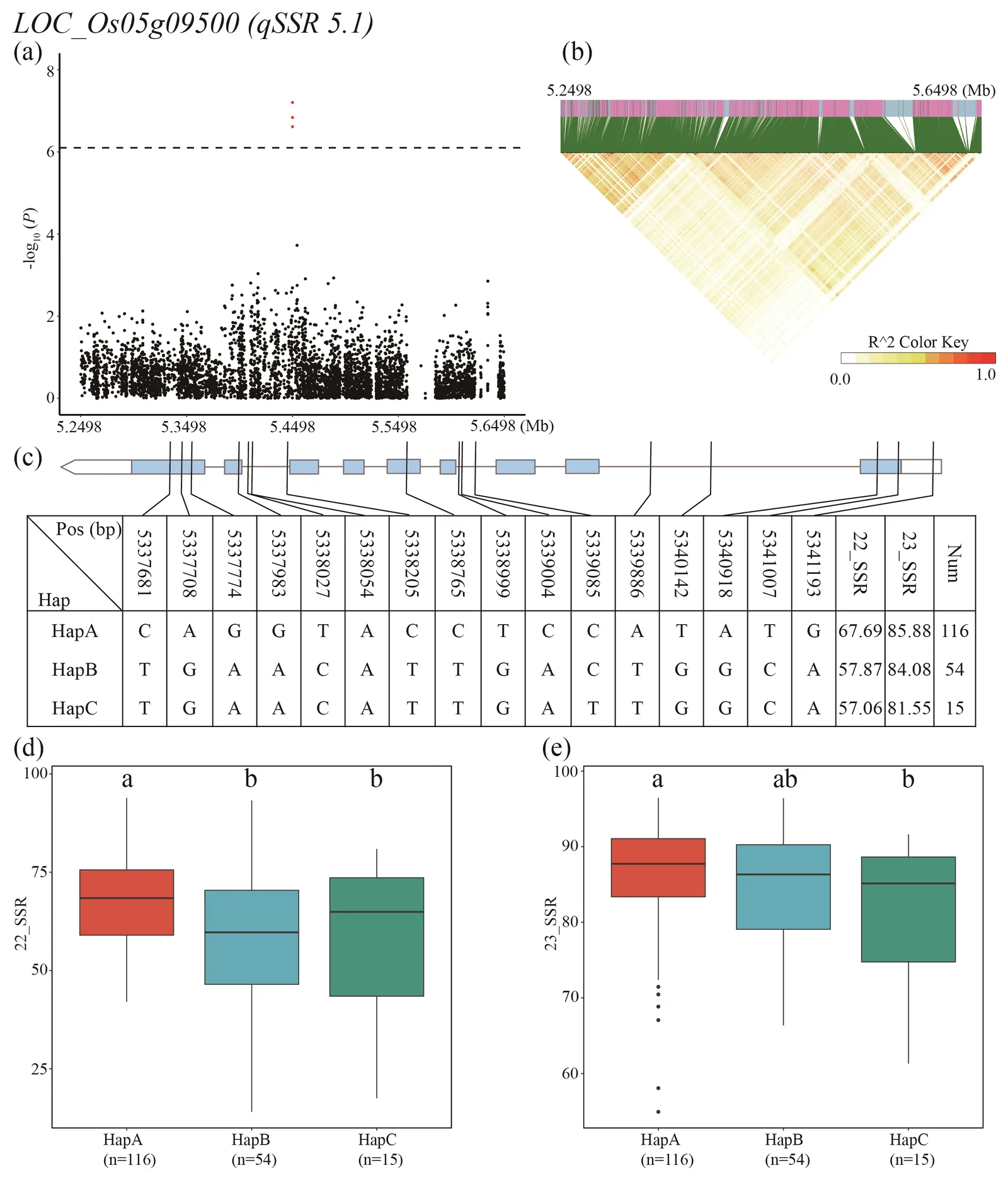
Haplotype analysis of *LOC_Os05g09500* associated with SSR. **(a)** Regional Manhattan plot of qSSR5.1; **(b)** Linkage disequilibrium (LD) heatmap of the candidate region; **(c)** gene structure and haplotype composition of *LOC_Os05g09500* haplotype region. Promoters are represented by white boxes, exons by blue boxes, and introns and intergenic regions by thin blue lines, collectively forming the gene structure of *LOC_Os05g09500*; **(d, e)** comparison of SSR among haplotypes in 2022 and 2023. Different letters indicate significant differences among haplotypes according to Tukey’s multiple comparison test (*p* < 0.05).

Expression analysis based on public RNA-seq data indicated that *LOC_Os05g09500* was expressed in reproductive tissues, particularly panicle and seed tissues (**Supplementary Table 5**). Its expression during grain development is consistent with the proposed role of hexokinase-mediated sugar sensing and carbohydrates metabolism in determining spikelet fertility and grain filling.

### 3.5 Candidate gene identification and haplotype analysis for plant height

For PH, 10 significant QTLs were identified. Candidate genes within all 10 PH associated QTL intervals were systematically annotated and corresponding gene information is provided in **Supplementary Table 4**. Within the *qPH3.1* interval (3.1-3.5 Mb), a total of 33 annotated genes were screened based on the functional annotation and sequence variation (**Supplementary Table 4**). Among these, *LOC_Os03g06580*, *LOC_Os03g06654*, *LOC_Os03g06705*, *LOC_Os03g06930*, and *LOC_Os03g06970* contained non-synonymous variants. Candidate prioritization was not based solely on non-synonymous variation; functional annotation, coding variation, haplotype-phenotype association, and publicly available expression information were considered together. Among the genes within *qPH3.1*, *LOC_Os03g06930*, annotated as *OsBLH6*, was prioritized for further analysis based on its transcription factor annotation, potential relevance to plant development, and informative sequence variation.

Haplotype analysis based on the polymorphisms within *LOC_Os03g06930* identified two major haplotypes (**Figure 5**). The mean PH values for HapA and HapB were 114.73 cm and 96.24 cm in 2022, and 122.23 cm and 102.98 cm in 2023, respectively, with the corresponding number of accessions being 99 and 85, respectively. Across the two year-environments, HapA consistently exhibited significantly higher PH values than HapB. The similar direction of haplotype effect in the two evaluated years supports further evaluation of HapA as a potentially favorable haplotype combination; however additional environments are required to assess its broader stability. Public expression data further showed that *LOC_Os03g06930* was expressed in shoot tissue at the 35-days seedling stage, with an expression value of 5.83138 (**Supplementary Table 5**), supporting its transcriptional activity during vegetative development. In brief, functional annotation, sequence variation, haplotype-phenotype associations and public expression evidence support the prioritization of the *LOC_Os03g06930* as a candidate gene associated with natural variation in PH.

**Figure 5:**
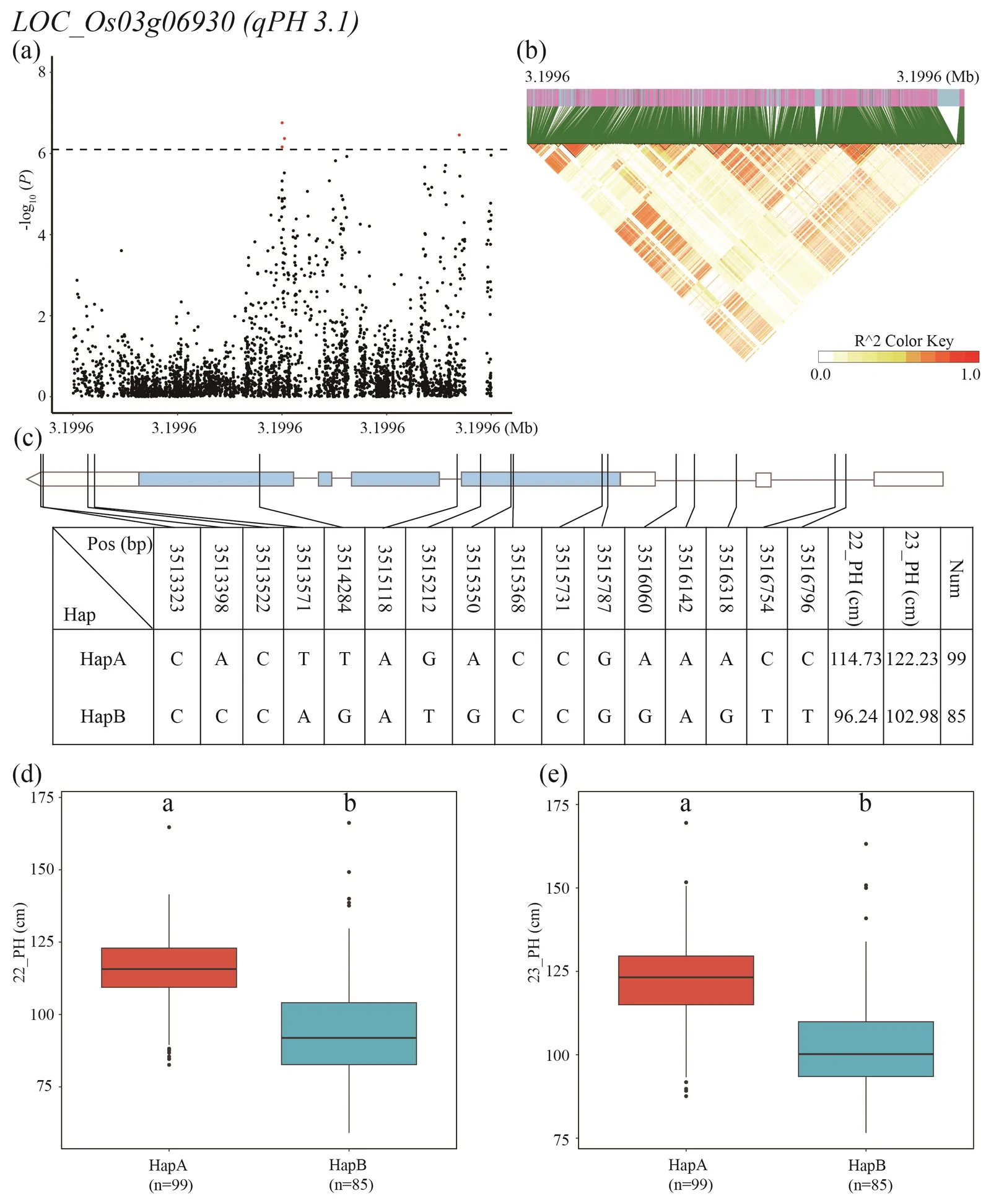
Haplotype analysis of *LOC_Os03g06930* associated with plant height. **(a)** Regional Manhattan plot of *qPH3.1;* **(b)** Linkage disequilibrium (LD) heatmap of the candidate region; **(c)** gene structure and haplotype composition of *LOC_Os03g06930* haplotype region. Promoters are represented by white boxes, exons by blue boxes, and introns and intergenic regions by thin blue lines, collectively forming the gene structure of *LOC_Os03g06930*; **(d, e)** comparison of PH among haplotypes in 2022 and 2023. Different letters indicate significant differences among haplotypes according to Tukey’s multiple comparison test (p < 0.05).

Expression analysis using public transcriptomic datasets showed that *LOC_Os03g06930* was expressed in shoot tissues (**Supplementary Table 5**), consistent with the proposed roles of the BLH family transcription factor. These expression data provide additional support for its possible contribution to PH variation.

### 3.6 Candidate gene identification and haplotype analysis for thousand-grain weight

For TGW, one significant QTL (*qTGW2.1*) was identified. Candidate genes within *qTGW2.1* interval were systematically annotated, and the corresponding gene information is provided in **Supplementary Table 4**. Within the *qTGW2.1* interval (4.5-4.9 Mb), 55 annotated genes were identified (**Supplementary Table 4**), Among these genes, 14 contained non-synonymous sequence variants. Candidate prioritization was not based solely on non-synonymous variation; functional annotation, coding variation, haplotype-phenotype association, and publicly available expression information were considered together. *LOC_Os02g09170*, was annotated as the sucrose phosphate synthase gene (*OsSPS2*), contained three non-synonymous variants and was prioritized for further analysis due to its biological relevance to carbohydrate metabolism and grain development.

Haplotype analysis based on the polymorphisms within *LOC_Os02g09170* classified rice accessions into five major haplotypes (**Figure 6**). In 2022, the mean TGW values of HapA, HapB, HapC, HapD, and HapE were 24.23 g, 23.60 g, 22.32 g, 25.71 g, and 22.96 g, respectively, with HapD exhibiting the significantly higher TGW than HapB, HapC, and HapE. In 2023, the corresponding TGW values were 24.96 g, 29.45 g, 27.43 g, 24.45 g, and 27.65 g, respectively, and HapB exhibiting significantly higher TGW than HapC and HapE. The five haplotypes were represented by corresponding numbers of accessions for these haplotypes were 83, 41, 30, 12, and 12 accessions, respectively. Although the ranking of haplotypes differed between years, significant TGW differences were consistently observed among haplotypes, indicating that genetic variation within *LOC_Os02g09170* is associated with natural variations in grain weight. Public expression data further showed that *LOC_Os02g09170* was expressed in developing seeds at 5 days after pollination, with an expression value of 10.36170 (**Supplementary Table 5**), supporting its potential involvement during grain development. In brief, functional annotation, sequence coding variation, haplotype-phenotype associations and public expression evidence support the prioritization of the *LOC_Os02g09170* as a candidate gene associated with natural variation in TGW.

**Figure 6:**
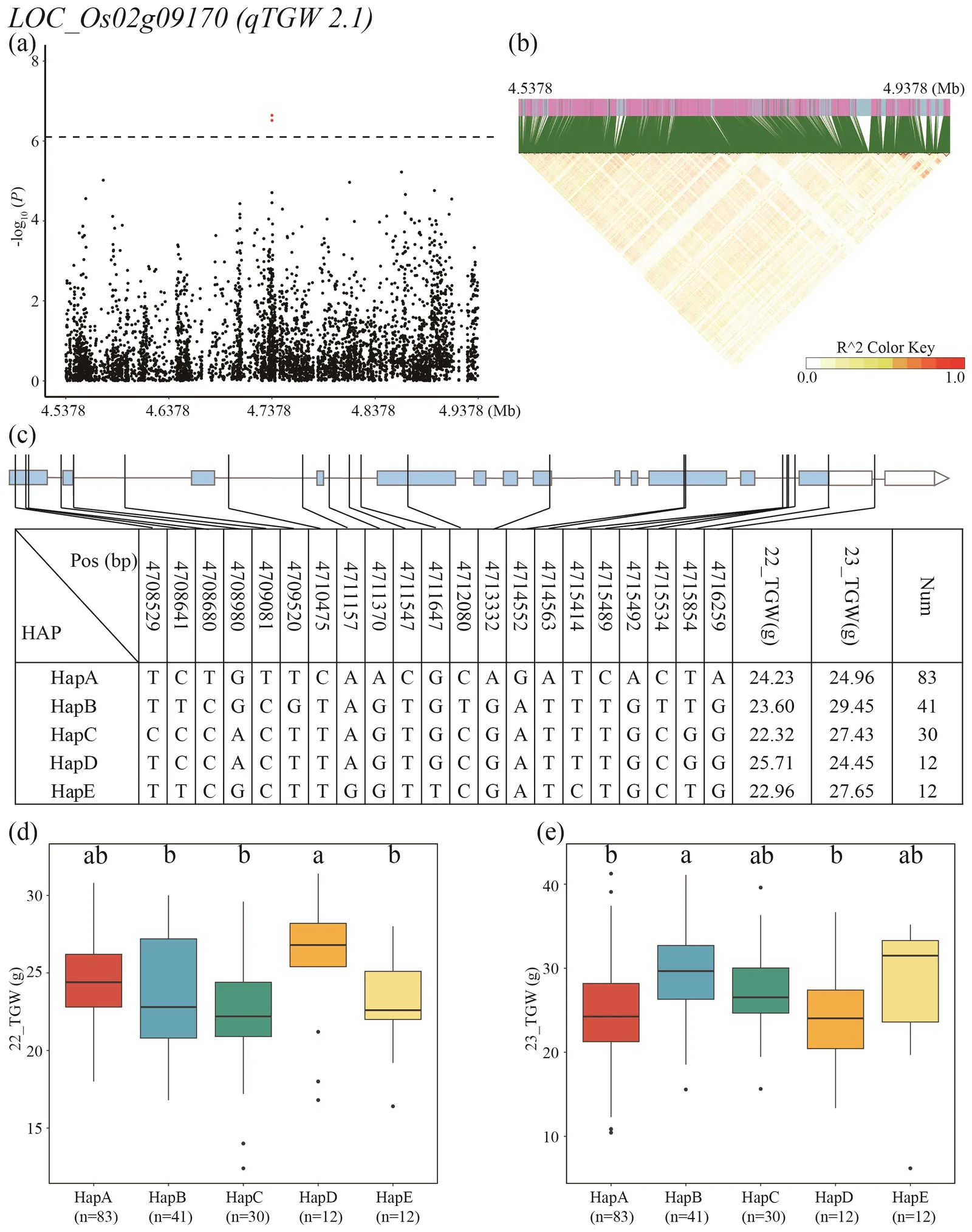
Haplotype analysis of *LOC_Os02g09170* associated with thousand grain weight. **(a)** Regional Manhattan plot of *qTGW2.1;* **(b)** Linkage disequilibrium (LD) heatmap of the candidate region; **(c)** gene structure and haplotype composition of *LOC_Os02g09170* haplotype region. Promoters are represented by white boxes, exons by blue boxes, and introns and intergenic regions by thin blue lines, collectively forming the gene structure of *LOC_Os02g09170*; **(d–e)** comparison of TGW among haplotypes in 2022 and 2023. Different letters indicate significant differences among haplotypes according to Tukey’s multiple comparison test (p < 0.05).

Public RNA-seq data revealed that *LOC_Os02g09170* showed expression in reproductive tissues (**Supplementary Table 5**). The expression profile of this gene is consistent with its proposed role in carbohydrate metabolism and grain filling regulation.

## 4 Discussion

Genome-wide association analysis identified 20 QTLs associated with GY, SSR, PH, and TGW. Among these loci 11 co-localized with previously reported genes or QTLs controlling yield-related traits, providing independent support for the reliability of the association signals identified in this study. The co-localization of the significant loci with well characterized genes involved in cytokinin signaling, flowering regulation, gibberellins response, and grain development suggests that the GWAs framework successfully captured biologically meaningful genomic regions underlying complex agronomic traits. However, because the present GWAS was based on BLUP values estimated from two year-environments, further evaluation across additional environments and formal QTL × environment interaction analysis will be required to determine the broader environmental reproducibility of these loci.

In the present study, GWAS was performed using BLUP values estimated across two growing seasons to minimize residual environmental variation and improve the accuracy of identifying genetic associations supported by integrated phenotypic information from two year-environments. However, because only two-year-environment were evaluated, and no year specific GWAS or QTL × environment interaction analysis was performed, the loci identified here should not be interpreted as environmentally stable QTLs. Similarly, resemblance in haplotype-phenotype associations between 2022 and 2023 provides supportive evidence of reproducibility across these two observations but does not establish stability across a broader range of environments. Future studies incorporating additional environments and years, together with year specific GWAS and formal QTL × environment analysis, will be needed to distinguish broadly reproducible loci from environment-specific loci associated with genotype × environment interactions.

Several identified QTLs overlapped with previously characterized genes known to regulate rice yield related traits. For example, *qGY 1.1* co-localized with *LOGL1* (Sandhu et al., 2024), a gene controlling GY that encodes a cytokinin-activating enzyme. Overexpression lines of *LOGL1* exhibited decreased tiller number, GNP, and GY. *qGY 7.1* col-localized with *Ghd7* (Xue et al., 2008) and *NAL8* (Chen et al., 2019), both of which grain productivity through regulation of flowering time, plant architecture, and panicle development. Near-isogenic lines (NILs) containing the wild-type *Ghd7* allele observed heading stage 23 days later than their parents Zhenshan-97 and showed a 50% increase in GY, accompanied by a series of other trait variations. In the *NAL8* mutant, auxin levels were elevated while cytokinin levels decreased and PH, flag leaf width, spikelet number, and TGW were reduced, leading to a significant decline in GY. *qGY 9.1*co-localized with *MOG1* (Han et al., 2024). Compared to the wild type, *MOG1* overexpression lines showed increases in PH, panicle length, primary and secondary branch number, GNP, grain length, grain width, TGW, and GY. No co-localized QTLs found for SSR, reported in previous literature. However, among the 10 QTLs for PH, 7 were co-localized. These included *DPF* (Yamamura et al., 2015), *D2* (Dong et al., 2016), and *OsRDR3* (Jha et al., 2021) in *qPH1.1* intervals. *D2* is involved in BR biosynthesis, and has recessive mutations that hinder BR biosynthesis, resulting in dwarfism. *SLRL1* (Itoh et al., 2005) was found in *qPH 1.2*; overexpression of SLRL1 leads to a dwarf phenotype, and its overexpression in the *slr1* mutant suppresses the slender phenotype of the mutant. *Os1BGlu4* (Xu et al., 2021) was identified in *qPH 1.3*; the mutant bh1 showed 13.17% and 12.45% reductions in grain length and width, respectively, compared to the wild type (WT), along with significantly reduced PH, TGW, and GNP. *OsINO80* (Li et al., 2018) was found in *qPH 3.2*. T-DNA insertion or RNAi knockdown mutants of *OsINO80*, as well as the heterozygous mutant osino80/+, exhibited typical GA-deficient phenotypes, including dwarfism, shortened cell length, delayed flowering, retarded seed germination, and impaired male gametophyte development. Additionally, *qPH 4.1*, *qPH 9.1* and *qPH 9.2* also contained genes like previously reported studies. For TGW, although only one QTL was mapped, we identified the gene *OsCAD2* (Wang et al., 2023) within *qTGW 2.1*. Compared to the wild type, the mutant *gh881* exhibited golden hulls and internodes, with extremely significant reductions in PH, total and filled GNP, SSR, and TGW, while the number of effective panicles per plant showed no difference. The gh881 phenotype was caused by variation in *OsCAD2*. The identification of these genes within significant GWAS intervals further supports the robustness of the association mapping results (Chen et al., 2022).

In this study, candidate genes underlying identified QTLs were prioritized using an integrated strategy combining genomic position, functional annotation, coding variation, haplotype-phenotype association and publicly available expression evidence. Across the 20 significant QTL intervals identified for GY, SSR, PH, and TGW, 532 annotated genes were catalogued (**Supplementary Table 4**). Among these genes, *LOC_Os09g28230* (*GID1L2*), *LOC_Os05g09500* (*OsHXK7*), *LOC_Os03g06930* (*OsBLH6*), and *LOC_Os02g09170* (*OsSPS2*) were prioritized because they showed multiple lines of supporting evidence, including trait relevant functional annotation, informative sequence variation, consistent haplotype effects across the two evaluated years, and expression patterns in biologically relevant tissues (**Supplementary Table 5**). For example, *LOC_Os09g28230* (*GID1L2*) was prioritized in *qGY9.2* because of its role as a gibberellin receptor, the presence of coding variation, consistent haplotype associated differences in grain yield and expression in reproductive tissues. Similarly, *LOC_Os05g09500* (*OsHXK7*), *LOC_Os03g06930* (*OsBLH6*), and *LOC_Os02g09170* (*OsSPS2*) were prioritized based on their functional relevance to sugar metabolism, plant development and carbohydrate metabolism, respectively, together with the supporting genetic and expression evidence. These results provide candidate genes that may contribute to natural variation in yield-related traits. However, these candidate genes should be considered as prioritized candidates rather than confirmed causal genes. Further functional validation such as gene editing, transgenic complementation, or detailed molecular characterization will be required to confirm their direct contributions to the observed phenotypic variations.

In this study, 265 accessions were genotyped, and a total of 4,454,137 single nucleotide polymorphisms (SNPs) were used for GWAS. We mapped 20 QTLs significantly associated with yield and identified 225 candidate genes within these intervals (**Table 1**; **Supplementary Table 3**), revealing the genetic complexity underlying yield-related traits in rice. Combined with haplotype analysis of candidate genes, functional annotation, and expression evidence, one prioritized candidate gene was identified for each trait: *LOC_Os09g28230*, *LOC_Os05g09500*, *LOC_Os03g06930*, and *LOC_Os02g09170* for GY, SSR, PH, and TGW, respectively, and their expression profiles were further evaluated using public RNA-seq datasets (**Supplementary Table 5**).

To further evaluate the biological relevance of these prioritized candidate genes, publicly available transcriptome data were integrated with haplotype analysis. The 4 candidate genes exhibited detectable expression in tissues and developmental stages related to yield formation, including panicle, seed, and shoot tissues (**Supplementary Table 5**). Although expression evidence alone cannot confirm gene function, these expression patterns provide additional support for their potential involvement in regulating yield-related traits. Further experimental validation using RT-qPCR, gene editing, and transgenic approaches will be required to confirm their causal roles.

The prioritized candidate gene for GY, *LOC_Os09g28230*, was annotated as GA receptor *GID1L2*. At position 17145876 in the exon region, a C-to-A mutation caused a Glu-to-stop codon change, leading to premature termination of protein translation. Haplotype analysis revealed that the mutation at 17145876 occurred in HapA, and the GY of HapA was significantly higher than that of the other two haplotypes. *GID1L2* is a member of the GA receptor GID1 family. Studies have shown that *OsGID1L2* is a protein containing a typical Abhydrolase_3 domain, which is widely involved in lipid metabolism/hormone signal transduction in plants (Hedden and Thomas 2012). Furthermore, IAA treatment for 2 hours triggered a significant 53-fold increase in *OsGID1L2* expression. It is hypothesized that the *GID1L2* protein may act as a downstream target of the auxin signaling pathway to participate in rapid stress-induced responses or integrate multiple plant hormone signals to regulate growth and metabolism (Ji et al., 2025). The significant phenotypic differences among haplotypes, together with known role of the GA signaling in biomass accumulation and reproductive development, suggest that *LOC_Os09g28230* is a promising candidate gene potentially contributing natural variation in GY.

The prioritized candidate gene for SSR, *LOC_Os05g09500*, harbored an A-to-G mutation at position 5337983 in the exon region, resulting in the loss of a stop codon and a change to Arg, which extended protein translation. The mutation at 5337983 occurred in HapA, and the SSR of HapA was significantly higher than that of the other two haplotypes. *LOC_Os05g09500* was annotated as cytosolic hexokinase. Hexokinase is a key enzyme in plant sugar metabolism and signal transduction; it not only catalyzes the phosphorylation of hexose to hexose-6-phosphate but also plays a vital role in sugar sensing and signaling (Cho et al., 2006). Grain filling is a critical process determining SSR and grain weight, in which sugar metabolism plays a core role. Past studies indicated that starch accumulation is closely related to the activity of sugar metabolism enzymes during rice grain development. In the early filling stage, the activities of enzymes such as ADPG pyro-phosphorylase and starch synthase rise rapidly, catalyzing the synthesis of starch from substrates like glucose. As a key upstream enzyme in sugar metabolism, *OsHXK7* influences the entire sugar metabolic network by regulating glucose phosphorylation. One of the most important functions of *OsHXK7* is regulating the carbon-nitrogen metabolic balance by forming a complex with the transcription factor ARE4. Nitrogen is a key nutrient element affecting rice yield, while carbon metabolites provide the carbon skeleton and energy for nitrogen assimilation. The *OsHXK7-ARE4* complex regulates nitrogen absorption by sensing glucose levels, achieving coordinated control of carbon and nitrogen metabolism. Efficient carbon and nitrogen utilization is essential for spikelet fertility and grain filling. Therefore, allelic variation affecting hexokinase-mediated sugar sensing and carbon-nitrogen coordination may contribute to variation in SSR performance. During the reproductive growth stage of rice, adequate nitrogen supply aids spikelet differentiation and grain development, while appropriate carbon sources provide energy and material foundations for these processes (Ma et al., 2023). Collectively, these findings support *LOC_Os05g09500* as a promising candidate gene associated with natural variation in SSR. It is noteworthy that phenotypic distribution of the SSR differed between the two growing seasons. In 2022, showed an approximately normal distribution, whereas in 2023 most accessions exhibited relatively higher SSR values, resulting in a right-skewed distribution. This difference is likely due to year-to-year environmental variation during reproductive stage. SSR is one of the most environmentally sensitive yield related traits and is strongly influenced by the environmental factors, particularly, temperature, humidity and other climatic conditions that affect pollen development, fertilization and grain filling. More favorable environmental conditions during the reproductive period in 2023 may promote successful fertilization and grain filling, leading to a higher overall SSR and reduced phenotypic variation compared with 2022. These observations further highlighted the importance of evaluating yield related traits across multiple environments and distinguish stable genetic effects from environmental influences.

The prioritized candidate gene for PH, *LOC_Os03g06930*, was annotated as bell-like homeodomain-containing transcription factor 6. At position 3515350 in the exon region, a G-to-A mutation caused an Arg-to-stop codon change, leading to premature termination of protein translation. The mutation at 3515350 occurred in HapA, and the PH of HapA was significantly higher than that of HapB. BLH family transcription factors are widely involved in fundamental developmental processes in plants, such as meristem maintenance and organ morphogenesis. Meanwhile, *LOC_Os03g06930* can synergize with transcription factors like *OsMYB55/61* to activate the promoters of key secondary cell wall biosynthesis genes such as *OsCAD2*, thereby promoting the synthesis and deposition of lignin and cellulose (Hirano et al., 2013). The thickness and strength of the secondary cell wall in stems are directly related to their supporting ability and lodging resistance, which in turn affect plant elongation and final height. The approximately18-19 cm difference observed between HapA and HapB across the two year-environments, suggests that allelic variations at *LOC_Os03g06930* may contribute substantially to PH variations within the studied population. These results indicate that *LOC_Os03g06930* is a promising candidate gene underlying natural variations in PH and plant architecture.

The prioritized candidate gene for TGW, *LOC_Os02g09170* was annotated as sucrose-phosphate synthase. Mutations occurred at positions 4708529, 4708641, and 4712080 in the exon region: a T-to-C mutation changed Leu1 to Arg; a C-to-T mutation changed Ala to Val; and a C-to-T mutation changed Ser to Leu. Studies have shown that increased expression of *OsSPS2* enhances the activity of sucrose-phosphate synthase in leaves, thereby promoting sucrose synthesis (Li and Cui, 2018). Sucrose synthesized in leaves is transported via the phloem to the “sink” organ the grain. Higher sucrose supply provides a richer carbon skeleton and energy for grain filling (Yan et al., 2022). The increased availability of carbon skeletons and energy during the filling stage allows for the synthesis and accumulation of more starch in the grain, which is the main component of rice dry matter, ultimately resulting in an increase in thousand-grain weight. Because grain filling depends largely on carbohydrates availability and starch accumulation, allelic variation affecting sucrose-phosphate synthase activity could influence final grain weight. Significant differences in TGW among *LOC_Os02g09170* haplotypes indicate the variation within the gene may contribute to grain weight determination, although performance ranking of different haplotypes differed across the two year-environments.

An important limitation of the present study is that phenotypic evaluation is comprised of only two year-environments at a single field location. This design enabled identification of genetic associations supported by phenotypic observations collected during two consecutive growing seasons; however, it does not provide comprehensive assessment of genotype × environment interactions or environmental stability, particularly for complex traits such as grain yield. Therefore, the identified loci and candidate genes represent genomic regions associated with variation in the evaluated traits within the studied population and two-year environments. Further validation across additional environments will be required to determine their broader environmental stability and breeding applicability.

The identification of candidate genes associated with GY, SSR, PH, and TGW provides valuable genetic resources for understanding the genetic basis of rice yield-related traits. The favorable haplotypes identified for *LOC_Os09g28230*, *LOC_Os05g09500*, and *LOC_Os03g06930* may serve as potential markers for future breeding applications after further validation. However, because the present study based on association and haplotype analysis, functional validation through gene-editing, transgenic experiments and expression analysis will be required to confirm their biological roles. Future studies integrating transcriptomic, metabolomic, and functional genomic approaches will help elucidate the molecular mechanism underlying yield formation in rice. In addition, although the SNP markers analyzed in this study were derived from high-quality whole genome resequencing data and subjected to stringent quality filtering, further experimental validation of representative GWAS-associated SNPs using targeted sequencing approaches, such as Sanger sequencing, would provide additional confirmation of these associations.

## 5 Conclusion

In this study, a genome wide association analysis was conducted using 265 rice accessions and 4454137 high-quality SNPs to investigate the genetic basis of 4 important yield related traits, including GY, SSR, PH, and TGW. A total of 20 significant QTLs were identified, of which 11 co-localized with previously reported genes or QTLs associated with rice yield -related traits, supporting the reliability of the association signals detected in this study. Through integrated analysis of candidate gene annotation, sequence variation, haplotype analysis, and expression evidence, four candidate genes were prioritized: *LOC_Os09g28230* (*GID1L2*) was prioritized as a candidate gene for GY, *LOC_Os05g09500* for SSR, LOC*_Os03g06930* for PH, and *LOC_Os02g09170* for TGW. Significant phenotypic differences among haplotypes suggest that natural allelic variation within these genomic regions may contribute to phenotypic diversity in rice yield-related traits. These findings provide valuable genetic resources for understanding the genetic basis of rice-yield related traits and may provide promising candidate targets for future validation and potential application in MAS and molecular breeding. However, because the present study was based on two year-environments at one location, additional multi-environment experiments and formal QTL × environment interaction analysis are required before environmental stability or broad breeding adaptation of the identified loci can be inferred. Further, functional validation through gene expression analysis, gene editing, and transgenic approaches would be required to clarify the molecular mechanisms underlying these associations and determine whether these candidate genes can be effectively utilized in rice improvement programs.

## Data Availability

Publicly available datasets were analyzed in this study. This data can be found here: https://www.ncbi.nlm.nih.gov/bioproject/PRJNA1141731/ Accession Number: PRJNA1141731.

## Supplementary material

Supplementary Figure 1 Chromosomal distribution of the identified QTLs and previously reported genes/QTLs associated with rice yield related traits. Vertical bars represent the 12 rice chromosomes. The positions of significant QTLs identified in the present study are indicated on the left side of each chromosome, whereas previously reported genes/QTLs co-localizing with the detected loci are shown on the right side. QTLs shown in the red represent newly identified loci that did not overlap with any previously reported genes/QTLs, while those shown in black were co-localized with previously reported genes/QTLs.

Supplementary Table 1 Information of 265 rice accessions with respect to 4 candidate genes.

Supplementary Table 2 Genome wide association analysis results for significant single nucleotide polymorphisms (SNPs) associated with grain yield (GY), seed setting rate (SSR), plant height (PH), and thousand grain weight (TGW) in the 265 rice accessions.

Supplementary Table 3 Information on non-synonymous single nucleotide polymorphisms (nsSNPs) occurring in exons related to yield-associated traits.

Supplementary Table 4 Comprehensive annotation of genes located within the candidate intervals of the significant QTLs identified for yield-related traits in rice.

Supplementary Table 5 Evidence supporting prioritization of candidate genes associated with yield related traits.
