# Supplementary material for: Genome wide association mapping and haplotype analysis identify genetic loci and candidate genes associated with yield related traits in rice (Oryza sativa L.)
Source: Front Plant Sci. 2026 Sep 14;17:1909754. doi: 10.3389/fpls.2026.1909754 (PMC13617088; doi:10.3389/fpls.2026.1909754)
Supplement: Supplementary file 1 [file DataSheet1.pdf]

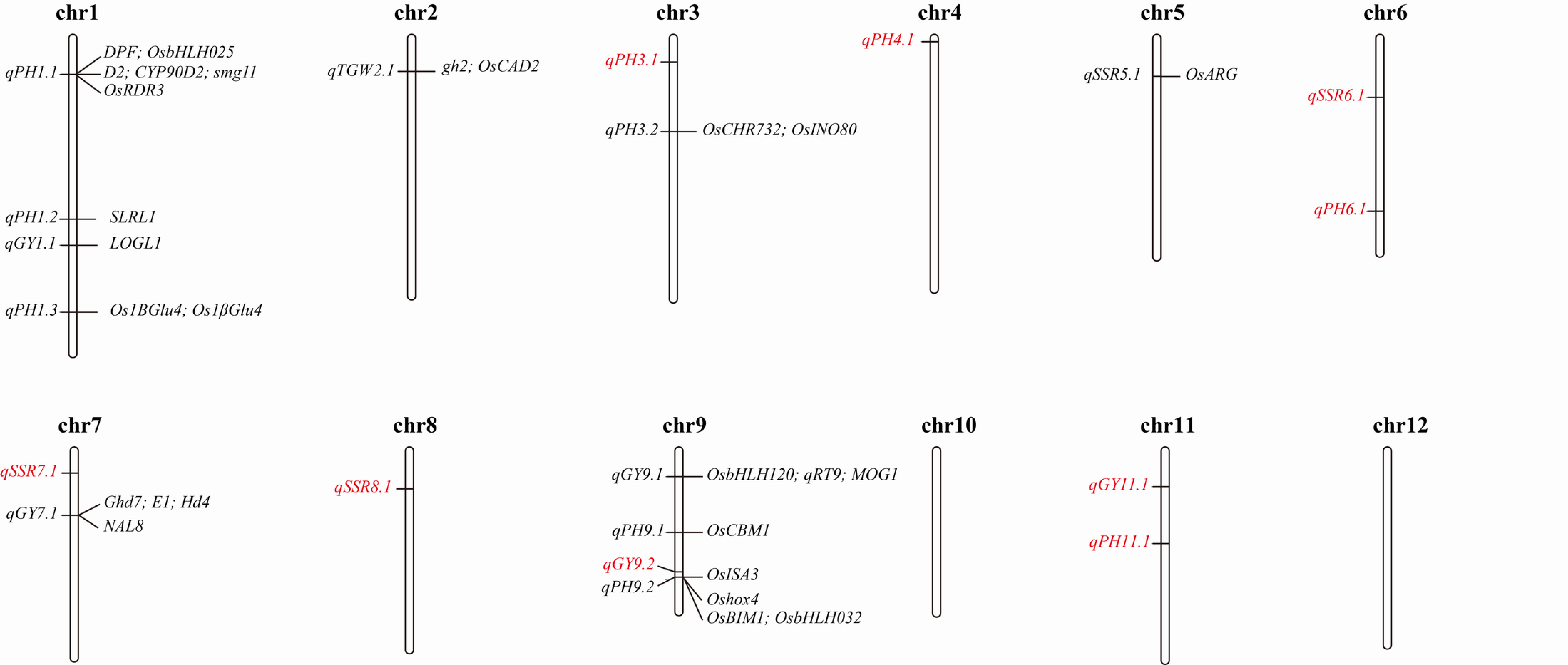
chr1
DPF; OsbHLH025
qPH1.1
D2; CYP90D2; smg11
OsRDR3
qPH1.2
SLRL1
qGY1.1
LOGL1
qPH1.3
Os1BGlu4; Os1βGlu4
chr2
qTGW2.1
gh2; OsCAD2
chr3
qPH3.1
qPH3.2
OsCHR732; OsINO80
chr4
qPH4.1
chr5
qSSR5.1
OsARG
chr6
qSSR6.1
qPH6.1
chr7
qSSR7.1
qGY7.1
Ghd7; E1; Hd4
NAL8
chr8
qSSR8.1
chr9
qGY9.1
OsbHLH120; qRT9; MOG1
qPH9.1
OsCBM1
qGY9.2
qPH9.2
OsISA3
Oshox4
OsBIM1; OsbHLH032
chr10
chr11
qGY11.1
qPH11.1
chr12
